# Illumina and Nanopore sequencing in culture-negative samples from suspected lower respiratory tract infection patients

**DOI:** 10.3389/fcimb.2024.1230650

**Published:** 2024-04-04

**Authors:** Lichao Ma, Chi Zhu, Tianli Yan, Yun Hu, Juan Zhou, Yajing Li, Furong Du, Jianping Zhou

**Affiliations:** ^1^ Department of Pulmonary and Critical Care Medicine, Wuxi Branch, Ruijin Hospital, Shanghai Jiao Tong University School of Medicine, Wuxi, Jiangsu, China; ^2^ State Key Laboratory of Neurology and Oncology Drug Development (Jiangsu Simcere Pharmaceutical Co., Ltd, Jiangsu Simcere Diagnostics Co., Ltd.), Jiangsu, China; ^3^ Nanjing Simcere Medical Laboratory Science Co., Ltd., Jiangsu, China; ^4^ Department of Respiratory and Critical Care Medicine, Ruijin Hospital, Shanghai Jiao Tong University School of Medicine, Shanghai, China; ^5^ Institute of Respiratory Diseases, Shanghai Jiao Tong University School of Medicine, Shanghai, China; ^6^ Shanghai Key Laboratory of Emergency Prevention, Diagnosis and Treatment of Respiratory Infectious Diseases, Shanghai, China

**Keywords:** metagenomic next-generation sequencing, bronchoalveolar lavage fluid, lower respiratory tract infection, Illumina, nanopore, culture-negative sample

## Abstract

**Objective:**

To evaluate the diagnostic value of metagenomic sequencing technology based on Illumina and Nanopore sequencing platforms for patients with suspected lower respiratory tract infection whose pathogen could not be identified by conventional microbiological tests.

**Methods:**

Patients admitted to the Respiratory and Critical Care Medicine in Shanghai Ruijin Hospital were retrospectively studied from August 2021 to March 2022. Alveolar lavage or sputum was retained in patients with clinically suspected lower respiratory tract infection who were negative in conventional tests. Bronchoalveolar lavage fluid (BALF) samples were obtained using bronchoscopy. Sputum samples were collected, while BALF samples were not available due to bronchoscopy contraindications. Samples collected from enrolled patients were simultaneously sent for metagenomic sequencing on both platforms.

**Results:**

Thirty-eight patients with suspected LRTI were enrolled in this study, consisting of 36 parts of alveolar lavage and 2 parts of sputum. According to the infection diagnosis, 31 patients were confirmed to be infected with pathogens, while 7 patients were diagnosed with non-infectious disease. With regard to the diagnosis of infectious diseases, the sensitivity and specificity of Illumina and Nanopore to diagnose infection in patients were 80.6% *vs*. 93.5% and 42.9 *vs*. 28.6%, respectively. In patients diagnosed with bacterial, *Mycobacterium*, and fungal infections, the positive rates of Illumina and Nanopore sequencer were 71.4% *vs*. 78.6%, 36.4% *vs*. 90.9%, and 50% *vs*. 62.5%, respectively. In terms of pathogen diagnosis, the sensitivity and specificity of pathogens detected by Illumina and Nanopore were 55.6% *vs*. 77.8% and 42.9% *vs*. 28.6%, respectively. Among the patients treated with antibiotics in the last 2 weeks, 61.1% (11/18) and 77.8% (14/18) cases of pathogens were accurately detected by Illumina and Nanopore, respectively, among which 8 cases were detected jointly. The consistency between Illumina and diagnosis was 63.9% (23/36), while the consistency between Nanopore and diagnosis was 83.3% (30/36). Between Illumina and Nanopore sequencing methods, the consistency ratio was 55% (22/42) based on pathogen diagnosis.

**Conclusion:**

Both platforms play a certain value in infection diagnosis and pathogen diagnosis of CMT-negative suspected LRTI patients, providing a theoretical basis for clinical accurate diagnosis and symptomatic treatment. The Nanopore platform demonstrated potential advantages in the identification of *Mycobacterium* and could further provide another powerful approach for patients with suspected *Mycobacterium* infection.

## Introduction

According to the WHO, lower respiratory tract infections (LRTIs) are an infectious disease with the highest mortality. LRTIs have been considered as a public health problem, which affect millions of people worldwide, causing thousands of deaths, and are treated with expensive medicines, such as antibiotics or palliative measuresThe top 10 causes of death (2023). The current conventional microbiological tests (CMTs) have a low positive rate for LRTI pathogen identification. It is reported that nearly 60% of patients with LRTIs died without a clear pathogen diagnosis (Besser et al., 2018). In this case, the early-time accurate identification of potential causative pathogens is crucial for LRTI treatment strategy.

In recent years, metagenomic next-generation sequencing (mNGS) has been widely used for pathogen detection in various clinical infectious diseases. It has been known that mNGS is independent of traditional culture and could detect a wide range of pathogenic microorganisms in a short time (about 30 h) (Besser et al., 2018; Li et al., 2021; Liu et al., 2023). Several studies have shown that mNGS has played an important role in the diagnosis of difficult and complex infectious diseases on various sample types, such as cerebrospinal fluid (CSF) in brain infections (Tian et al., 2023; Xiang et al., 2023; Ramachandran et al., 2024), bronchoalveolar lavage fluid (BALF) in LRTIs (Chen et al., 2020; Wu et al., 2020), and blood specimen in bloodstream infections (BSIs) (Crawford et al., 2020; Geng et al., 2021; Hoenigl et al., 2023). mNGS provides an alternative diagnosis method for suspected infectious patients, especially for those who have severe clinical symptoms without positive microbiological test results.

As sequencing technologies keep evolving, Nanopore sequencing technology has been put into clinical applications due to its long reads and shorter turnaround time (TAT). Meanwhile, there is no need for polymerase chain reaction (PCR) amplification during sequencing library construction. A Nanopore sequencer could avoid the preference and errors induced by PCR and further shorten the detection time in various kinds of body fluids (Zhu et al., 2021).

Currently, there are still few studies on the way to explore the clinical value of mNGS in LRTIs with negative CMTs. This study aims to use metagenomic sequencing based on both Illumina and Nanopore sequencing platforms. We focused on patients with suspected LRTIs without positive CMT results and evaluated the potential value of mNGS in the identification of microbiological tests.

## Materials and methods

### Study population and design

Patients admitted to the Respiratory and Critical Care Medicine in Shanghai Ruijin Hospital were retrospectively studied from August 2021 to March 2022. Cases were enrolled according to the following inclusion criteria: i) patients with symptoms like cough, expectoration, hemoptysis, chest pain, and fever; ii) patients with radiological images (chest X-ray or CT scanning) showing pulmonary shadow; iii) patients with a suspected infection causing the conditions described meeting criteria i) and/or ii); iv) negative CMT results; and v) sufficient BALF collected for laboratory testing. The exclusion criteria were as follows: i) patients with positive CMT results; ii) patients who did not sign the informed consent or refused to participate; iii) insufficient samples collected for testing; and iv) missing clinical information. This study was approved by the Ethics Committee of Ruijin Hospital, Shanghai Jiao Tong University School of Medicine (AF0406), and the informed consent forms were signed by the patients or surrogates.

### Sample collection and CMTs

BALF samples were obtained using bronchoscopy. Sputum samples were collected, while BALF samples were not available due to bronchoscopy contraindications.

The BALF and sputum samples were arranged for conventional microbiological tests and Illumina and Nanopore sequencers. In this study, CMT included bacteria and fungi culture and smear, G/GM test for fungi, mycobacterial culture, T-SPOT, and Xpert for MTB/NTM diagnosis. The CMT results of all enrolled patients were negative. The final clinical diagnosis was decided by two experienced doctors based on clinical records, radiological images, histopathological analysis, and treatment outcomes.

### DNA extraction

Sputum samples need to be liquefied first. Before DNA extraction, an aliquot of 1–2 ml of BALF or sputum specimen was conducted to break the cell wall using FastPrep Homogenizers (MP Biomedicals, CA, United States). Then, DNA was extracted from the homogenization using a microsample genomic DNA extraction kit (Tiangen, Beijing, China) according to the manufacturer’s specifications. Next, the host DNA was removed using saponin, as described previously (Charalampous et al., 2019). Finally, DNA concentrations were determined using Qubit 4.0 (Thermo Fisher Scientific Inc., Waltham, MA, United States).

### Library construction, sequencing on NextSeq 550Dx, and bioinformatic analysis

Approximately 200–300 bp of DNA fragments were obtained by enzymolysis. Then, to construct the sequencing library, DNA fragmentation, end repair, adapter ligation, and PCR amplification were conducted. Quality control of the DNA library was detected using Agilent 2100 Bioanalyzer (Agilent Technologies, Santa Clara, CA, United States). The qualified library was sequenced on NextSeq 550Dx sequencers (Illumina, CA, United States) using a 75-cycle single-end sequencing strategy, resulting in roughly 20 million reads per sample.

Clean reads were obtained by filtering out the short (<50 bp) and low-quality reads and removing duplicate reads using fastp. Reads mapped to the human genome (GRCh38) were removed using Burrows–Wheeler Alignment (VanRaden et al., 2019). After removing human sequences, the remaining reads were considered to potentially have microorganisms.

We built an in-house infectious pathogen database selected from the NCBI Nucleotide and Genome databases. Microbial classification was performed using Kraken2 and verified by BLAST. All the mapped reads were processed for taxonomy annotation, genome coverage calculation, and abundance calculation with in-house scripts. Then, the organisms were reported as pathogens detected by the metagenomic sequencing pipeline.

### Library construction, sequencing on GridION X5, and bioinformatic analysis

The sequencing library of the extracted DNA was constructed using the Rapid PCR Barcoding Kit (Oxford Nanopore Technologies, UK) following the recommended protocol, and sequencing was performed on GridION X5 (Oxford Nanopore Technologies, UK). Four hours of sequencing was needed.

Raw data were acquired using the MinKNOW software. Reads less than 500 bp and with a mean score lower than 6 were removed. The read alignment and pathogen detection procedures were performed the same as analyzing the data obtained from the Illumina sequencer.

### Diagnosis of pathogens

Bacteria (excluding mycobacteria), viruses, and parasites: A microorganism is considered clinically important when its coverage is 10 times higher than the coverage of any other microorganism. Fungi: They are considered clinically significant organisms when the coverage is 5 times higher than any other fungus. Mycobacteria: The detection of mycobacteria with a sequence number greater than or equal to 1 required a clinical note (Guo et al., 2021). The etiological diagnosis is based on the pathogens determined by a chief physician combined with various methods and the application of the corresponding drug treatment.

### Statistical analysis

SPSS 22.0 statistical software was used for data analysis, and GraphPad Prism 8 was used for plotting. The counting data were expressed as the number of cases (percentage) [*n* (%)], and the data between groups were compared by chi-square test or Fisher’s exact test. Clinical etiological diagnosis was used as the reference standard to evaluate the diagnostic efficacy of the two methods. To evaluate the diagnostic efficacy, 2 × 2 contingency tables and receiver operating characteristic (ROC) curves were used. The McNemar test and Kappa consistency analysis were used for the comparison of the positive rates between the two methods. A two-tailed value of *p <*0.05 represented significant differences.

## Results

### Characteristics and samples

From August 2021 to March 2022, 38 patients with suspected LRTI were enrolled in this study, consisting of 24 men and 14 women, after excluding 7 cases with incomplete clinical information. The median age of patients was 55 (36, 63) years. A total of 36 BALF samples and 2 sputum samples were subjected to mNGS based on Illumina and Nanopore sequencing platforms. The prognosis of one patient was not improved due to the progression of lung cancer and MTB infection, and the outcomes of the other patients were improved. Specific baseline data are shown in **Table 1**.

**Table 1:** Characteristics of the enrolled patients.

Characteristic	*n* (%)/IQR
Male	24 (63.2%)
Age, years	55 (36, 63)
Antibiotic use before tests	23 (55.3%)
Comorbidity	
Diabetes	4 (10.50%)
Hypertension	6 (15.8%)
Cardiovascular disease	4 (10.50%)
History of cancer	4 (10.5%)
COPD	1 (2.6%)
Hospital stay	8 (6, 13)
Prognosis	
Improved	37 (97%)
Not improved	1 (3%)
CURB-65 score	
0	25 (66%)
1	10 (26%)
2	2 (5%)
3	1 (3%)

### Consistency of species detected by Illumina and Nanopore

**Figure 1:**
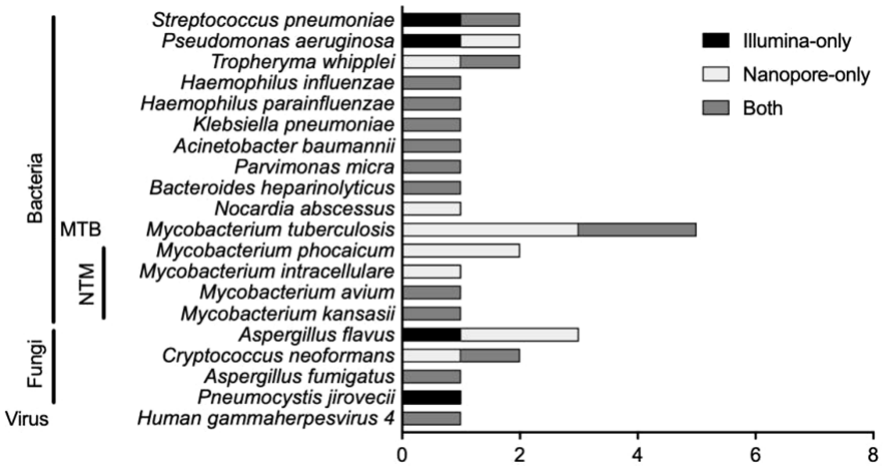
Pathogen comparison in suspected LRTI patients detected by Illumina and Nanopore.

There were 20 types of infectious pathogens detected by both methods and verified by clinical physicians, consisting of 15 kinds of bacteria [including 1 *Mycobacterium tuberculosis* (MTB) and 4 non-tuberculous mycobacteria (NTM)], 4 kinds of fungi, and 1 virus (**Figure 1**). For bacterial detection, except for MTB and NTM, *Streptococcus pneumoniae* was the most detected bacteria, and *Pseudomonas aeruginosa* and *Tropheryma whipplei* ranked second and third. The Illumina sequencer identified MTB in 2 cases, while Nanopore sequencing detected MTB in 5 cases. For NTM identification, there were 4 types of NTM identified by both methods, namely, *Mycobacterium phocaicum*, *Mycobacterium intracellulare*, *Mycobacterium avium*, and *Mycobacterium kansasii*. *Mycobacterium phocaicum* and *M. intracellulare* were detected only in the Nanopore sequencing method, and the other two types of NTM were detected by both sequencing methods. *Aspergillus flavus* was the most frequently detected species in fungi.

### Infection and etiology diagnosis

**Figure 2:**
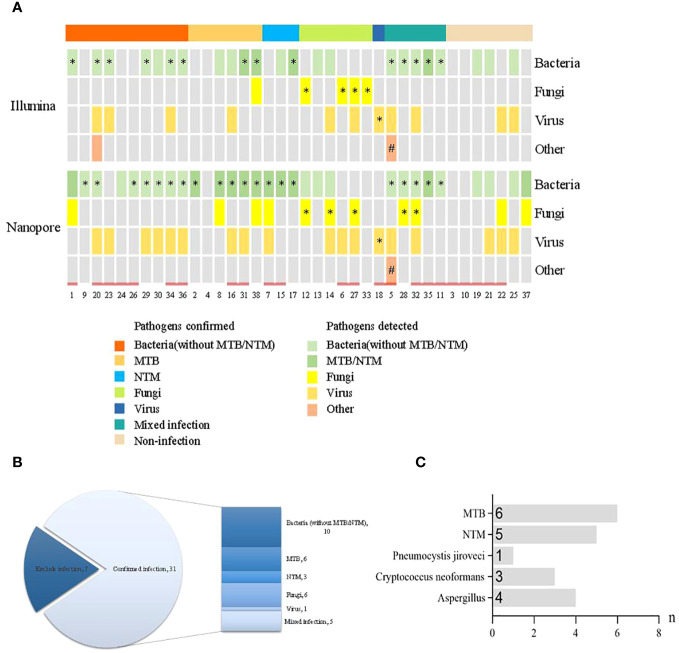
Distribution of pathogen detection by the Illumina and Nanopore sequencers compared with clinical infection and etiology diagnosis. **(A)** Distribution of pathogens detected by the two methods. **(B)** Distribution of infection diagnosis in the enrolled patients. **(C)** Distribution of specific pathogens confirmed by clinical infection diagnosis. * The results of pathogens detected were consistent with clinical diagnosis. # The clinical diagnosis was parasitic infection but the pathogen was not detected. = History of antibiotic use prior to pathogen detection.

The distribution of pathogen detection by the Illumina and Nanopore sequencers compared with clinical Infection and etiology diagnosis is shown in **Figure 2A**. According to the infection diagnosis, 31 patients were confirmed to be infected with pathogens, while 7 patients were diagnosed with non-infectious disease. Based on the patient’s perspective, there were 19 bacterial infections (6 with MTB and 3 with NTM), 6 fungal infections, 1 virus infection, and 5 mixed infections among all 31 infectious patients (**Figure 2B**). Based on the pathogen perspective, 15 patients (excluding *Mycobacterium* and including patients with mixed infection) had bacterial infection, 11 had *Mycobacterium* infection, 8 had fungal infection, 1 case had parasitic infection, and 1 case had virus infection (**Figure 2C**).

### Comparison of the diagnostic performance in infection diagnosis and pathogen diagnosis

**Figure 3:**
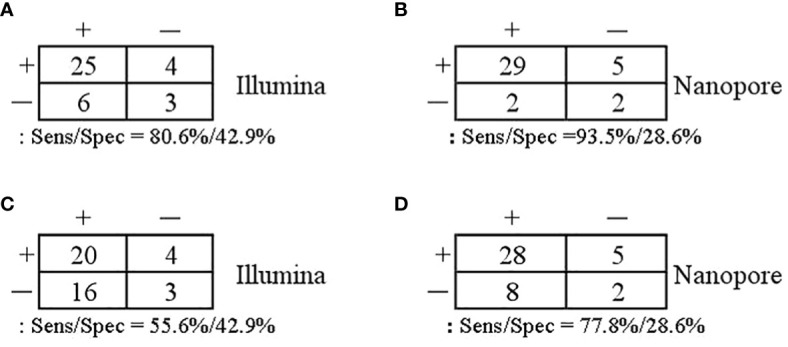
Comparison of the diagnostic performance in patients and pathogens. **(A, B)** Patients confirmed/excluded infection as a reference standard. **(C, D)** Accurate detection of pathogens as a reference standard.

**Figure 4:**
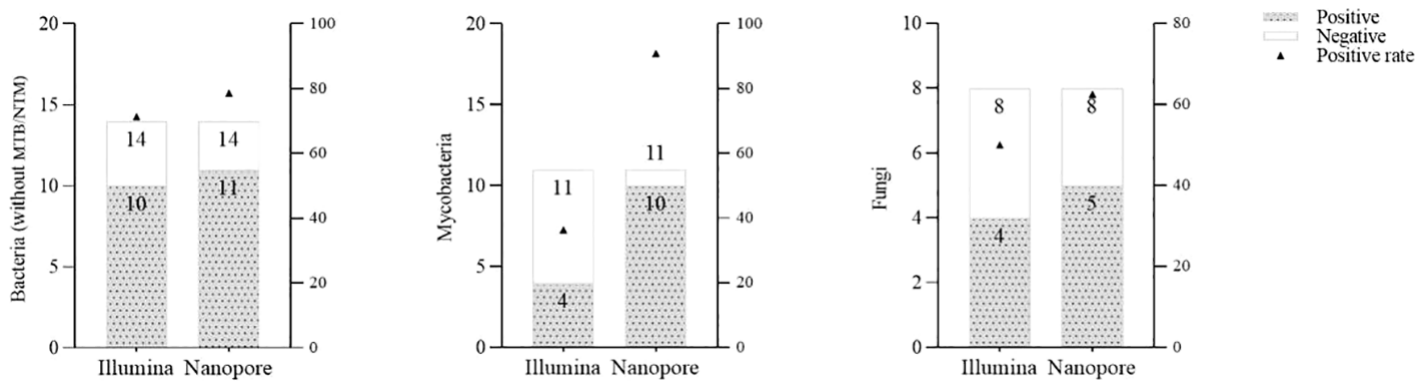
Comparison of the positive rates by the Illumina and Nanopore sequencers in different types of pathogens.

With regard to the diagnosis of infectious diseases, the sensitivity and specificity of Illumina and Nanopore to diagnose infection in patients were 80.6% *vs*. 93.5% and 42.9 *vs*. 28.6%, respectively. In terms of pathogen diagnosis, the sensitivity and specificity of pathogens detected by Illumina and Nanopore were 55.6% *vs*. 77.8% and 42.9% *vs*. 28.6%, respectively (**Figure 3**). In patients diagnosed with bacterial, *Mycobacterium*, and fungal infections, the positive rates of the Illumina and Nanopore sequencers were 71.4% *vs*. 78.6% (*P* = 1, Kappa = 0.05, *P* = 0.8), 36.4% *vs*. 90.9% (*P* = 0.01, Kappa = 0.4, *P* = 0.002), and 50% *vs*. 62.5% (*P* = 0.7, Kappa = 0.5, *P* = 0.02), respectively (**Figure 4**).

### Antibiotic use and pathogen detection

**Figure 5:**
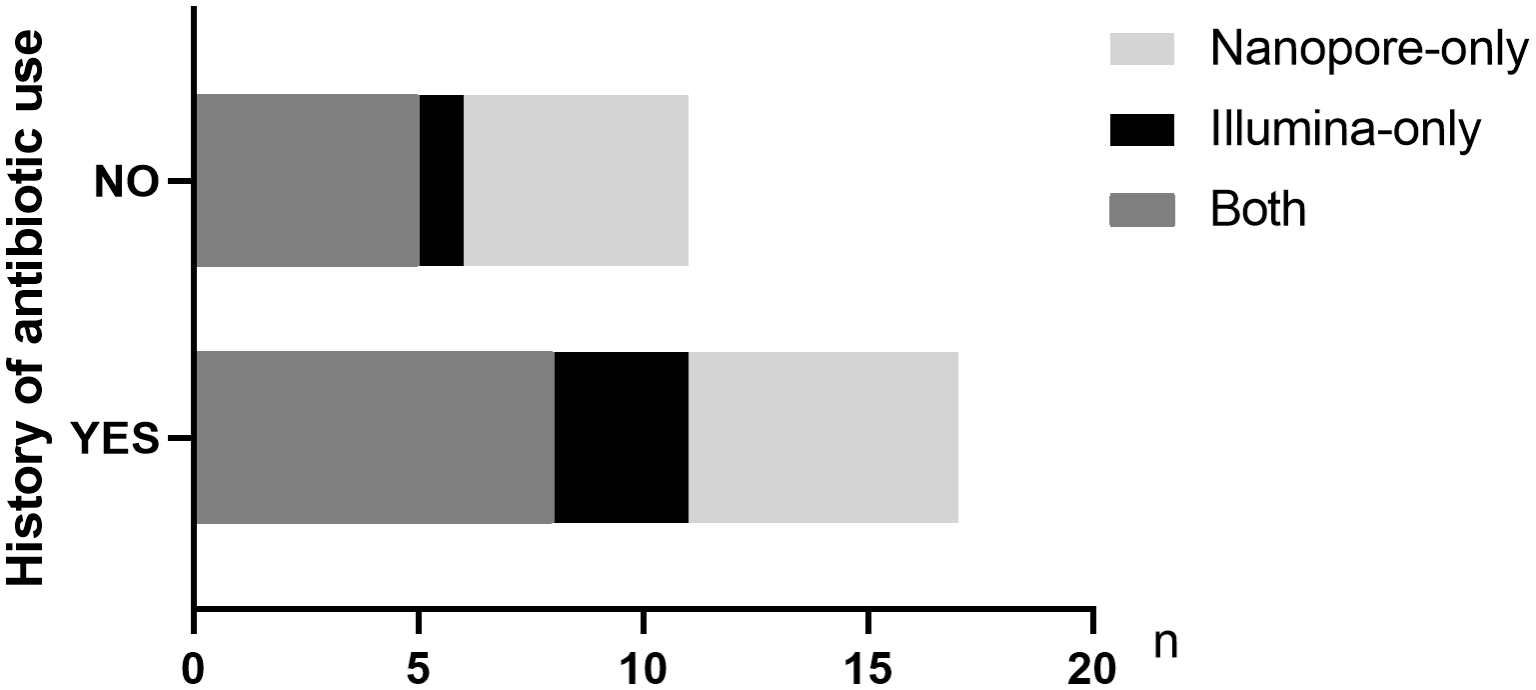
Distribution of pathogens detected by the two metagenomic sequencing methods in infected patients with or without antibiotic history.

Twenty-three out of 38 patients had a history of antibiotic use prior to pathogen detection. Among the 31 patients finally diagnosed with infectious diseases, 18 patients had a history of empirical antibiotic use. Among the patients treated with antibiotics in the last 2 weeks, 61.1% (11/18) and 77.8% (14/18) cases of pathogens were accurately detected by Illumina and Nanopore, respectively (*P* = 0.05), among which 8 cases were detected jointly. In patients without a history of antibiotic use, the Illumina and Nanopore detection rates were 46% (6/13) and 77% (10/13), respectively (*P* = 0.02) (**Figure 5**).

### Consistency between Illumina and Nanopore sequencing and etiological diagnosis

**Figure 6:**
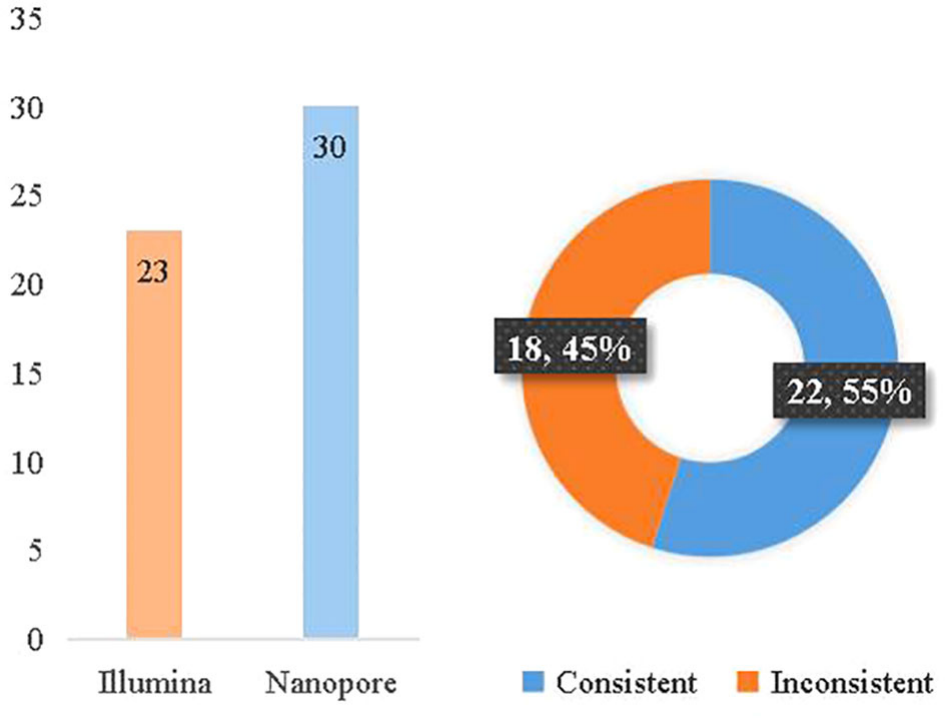
Consistency between mNGS and etiological diagnosis.

According to the clinical diagnosis, 31 patients were considered to have infectious diseases with 36 pathogens confirmed, while 7 patients had non-infectious diseases. The consistency between Illumina and diagnosis was 63.9% (23/36), while the consistency between Nanopore and diagnosis was 83.3% (30/36). Between Illumina and Nanopore sequencing methods, the consistency ratio was 55% (22/42) based on pathogen diagnosis (**Figure 6**).

## Discussion

Metagenomic next-generation sequencing, as a novel diagnosis tool, has been widely used in the diagnosis of infectious diseases. Its advantages compared with the traditional culture are obvious: nearly all microorganisms could be detected only once with unbiased, higher sensitivity, shorter TAT, and without any assumption. In this way, overuse and misuse of antibiotics as well as antibiotic resistance could be reduced. Nanopore sequencing technology, known as the fourth-generation sequencing method, had high accuracy and shorter TAT, making it ideal for infectious disease diagnosis (Harris et al., 2024).

In this study, we retrospectively analyzed the metagenomic sequencing results (including Illumina and Nanopore sequencing results) of 38 suspected LRTI patients, in which 31 patients were diagnosed with pathogen infection. From the perspective of overall clinical etiological diagnosis, the pathogens not detected by CMT are still mainly bacterial, especially *Mycobacterium*. This is related to the rigor of the conventional culture of *Mycobacterium* and the limitations of traditional methods. Overall, metagenomic sequencing accurately detected the pathogens in 29 out of 31 patients. Obviously, metagenomic sequencing has significant sensitivity in identifying these infections that CMT cannot recognize (Illumina: 80.6%, Nanopore: 93.5%). Illumina and Nanopore also show some advantages when it comes to accurately identifying difficult pathogens. The sensitivity and specificity of pathogens detected by Illumina and Nanopore were 55.6% *vs*. 77.8% and 42.9% *vs*. 28.6%, respectively. Furthermore, we analyzed the positive rates of the two platforms between different types of pathogens. The positive rates of bacteria and fungi were similar, but Nanopore had some advantages in the identification of *Mycobacterium*. Consistent with our previous studies (Zhang et al., 2022), Nanopore showed potential performance in the identification of mycobacteria in both CAP patients and CMT-negative patients. This is related to the more targeted pathogen detection conducted in the Nanopore test flow for microorganisms with difficulty in breaking walls during nucleic acid extraction, such as *M. tuberculosis* and *Aspergillus fumigatus*. Furthermore, the Nanopore platform employs a targeted enrichment method to amplify TB/NTM signals, thereby enhancing the detection rate of *Mycobacterium*. This could potentially account for the observed higher sensitivity of *Mycobacterium* detection using Nanopore technology. However, despite enhancing the sensitivity of detection, the Nanopore pipeline did not demonstrate a distinct advantage in terms of specificity. In summary, while Nanopore offers certain conveniences in pathogen diagnosis, its overall value in pathogen detection necessitates further prospective evaluation with large sample sizes and head-to-head comparisons.

Due to the high negative result ratio and long TAT of CMT, it is often suggested to empirically use antibiotics for suspected LRTI patients. The use of empirical antibiotics has a negative effect on the detection of pathogens by traditional methods, especially culture. In our study, 61% (23/38) of patients had been using antibiotics within 2 weeks before sampling. Among the patients with a history of antibiotic use who were finally diagnosed with pathogen infection, 94.4% (17/18) of patients were detected by metagenomic sequencing. It is worth noting that the CMT of these patients was negative, and the clinical application of mNGS provides a theoretical basis for the accurate treatment of the patients. In general, pathogen detection whether using Illumina sequencing or Nanopore sequencing was not affected in patients who have a history of antibiotic use. Similar results have been reported regarding mNGS being less influenced by prior antibiotic exposure (Miao et al., 2018; Shangguan et al., 2023).

Tuberculosis (TB) is one of the leading causes of death worldwide, with high mortality in a single infectious pathogen (Harding, 2020). MTB is the major causative pathogen for TB. NTM, belonging to the same *Mycobacterium* genus, can cause similar clinical performance but need entirely different treatments, which is hard but vital to distinguish them clinically. Quite a few studies reported that mNGS could be a powerful diagnostic tool for MTB detection (Jin et al., 2020; Gu et al., 2021; Liu et al., 2021; Xu et al., 2022). In this study, five out of six patients with clinically confirmed TB infection were detected by mNGS. All the NTM pathogens were detected by Nanopore sequencing methods. Of the total 11 confirmed cases of *Mycobacterium* infection, 10 were detected by Nanopore and 4 by Illumina.

Nowadays, different sequencing platforms have been used for metagenomic sequencing of clinical samples (Charalampous et al., 2019). Nanopore sequencing technology, known as rapid sequencing and real-time analysis, has potential advantages when applied in clinical settings. In addition, Nanopore sequencing methods have been optimized in studies and the total sequencing time has become shorter (Charalampous et al., 2019; Zhu et al., 2021). Furthermore, Nanopore metagenomic sequencing has been used for infectious pathogen detection.

Wang et al. compared the detection accuracy of mNGS based on BGI-seq and Nanopore sequencing methods for culture-negative sputum samples from patients with severe pneumonia. mNGS and Nanopore both reported major pathogens with approximately 60% consistency (Wang et al., 2020). Similarly, the consistency between Illumina and Nanopore sequencing was 55%, and Nanopore sequencing showed a higher rate of consistency in the final diagnosis.

This study reports the practical application of dual-platform metagenomic sequencing in CMT-negative patients with suspected LRTI. However, there were still several limitations in this study. First, the sample size was limited and only one case was ultimately diagnosed with virus function, which would affect the accuracy of the evaluation of the metagenomic sequencing performance on both sequencing platforms. Second, the sample types vary, including BALF and sputum. Taking into account the actual condition of the patients, BALF was not as convenient to obtain as sputum. In addition, the targeted enrichment process was additionally implemented on the Nanopore platform for assessing the diagnostic value of both methods; thus, the head-to-head comparison lacked strictness. For further studies, large-scale multicenter prospective studies are required to assess the value of metagenomic Nanopore sequencing. In addition, the effect of sample type needs to be given sufficient consideration.

In summary, both platforms play a vital role in infection diagnosis and pathogen diagnosis of CMT-negative suspected LRTI patients, providing a theoretical basis for clinical accurate diagnosis and symptomatic treatment. The Nanopore platform demonstrated potential advantages in the identification of *Mycobacterium* and could further provide another powerful approach for patients with suspected *Mycobacterium* infection.

## Data availability statement

The datasets presented in this study can be found in online repositories. The names of the repository/repositories and accession number(s) can be found at https://ngdc.cncb.ac.cn/omix/, OMIX006085.

## Ethics statement

The studies involving humans were approved by Ethics Committee of Ruijin Hospital, Shanghai Jiao Tong University School of Medicine. The studies were conducted in accordance with the local legislation and institutional requirements. The participants provided their written informed consent to participate in this study.

## Author contributions

LM, CZ participated in writing the manuscript. TY and YH conduct the study design. JZ, YL provide clinical information and case data. FD take part in a discussion and analysis of data. JiZ were in charge of the whole research project. All authors contributed to the article and approved the submitted version.
